# Total Antioxidant Status in Critically Ill Patients with Traumatic Brain Injury and Secondary Organ Failure—A Systematic Review

**DOI:** 10.3390/diagnostics14222561

**Published:** 2024-11-15

**Authors:** Ewa Rynkiewicz-Szczepanska, Urszula Kosciuczuk, Mateusz Maciejczyk

**Affiliations:** 1 Department of Anaesthesiology and Intensive Therapy, Medical University of Bialystok, Kilinskiego Street 1, 15-276 Bialystok, Poland; ewaryn@wp.pl; 2 Department of Hygiene, Epidemiology, and Ergonomics, Medical University of Bialystok, Kilinskiego Street 1, 15-276 Bialystok, Poland; mat.maciejczyk@gmail.com

**Keywords:** antioxidants, brain, oxidative stress

## Abstract

**Introduction:** The available literature indicates that oxidant–antioxidant imbalance plays a significant role in the pathophysiology of traumatic brain injury and the subsequent secondary organ dysfunctions. However, there is a lack of studies summarizing the knowledge in this area, and no clear guidelines exist regarding the use of biomarkers of oxidative stress as diagnostics tools. **Methods:** The present work aims to provide a systematic review of the literature on the use of total antioxidant capacity (TAC) assays in predicting the outcomes of traumatic brain injury (TBI). A literature search was conducted up to 1 September 2024, according to the Preferred Reporting Items for Systematic Reviews and Meta-Analyses (PRISMA 2020) guidelines, using the PubMed and Scopus databases. Based on the inclusion criteria, 24 studies were used for the final review. **Results:** Promising data indicate that TAC assays are useful in predicting 30-day mortality and neurological outcomes. Moreover, they correlate with radiological findings on CT scans in brain injury and the clinical classifications of injuries, as well as the parameters of organ failure. **Conclusions:** Total antioxidant capacity assays can be used to assess the extent of brain damage and prognosticate general vital functions. Future experiments should include long-term randomized clinical trials on larger populations of TBI patients.

## 1. Introduction

A total of 55% of traumatic brain injuries (TBIs) become life-threatening early on and require treatment in an intensive care unit (ICU) [1,2,3]. The mortality rate for TBI within the first 24 h is generally 8–10%, with rates of about 15% in the 15–24 age group and 71% among elderly people over the age of 85 years [4,5,6,7,8]. Moreover, TBIs are dynamic, and within 48 h, up to 46% of the victims develop secondary severe brain injuries [5,9,10,11]. Moreover, poor neurological outcomes, along with a predisposition to neurodegenerative changes, are significant consequences of brain trauma [12,13,14]. The disturbance of consciousness, airway obturation, and a lack of protective reflexes increase the risk of aspiration pneumonia and acute respiratory distress syndrome (ARDS). Sudden increases in intracranial pressure (ICP) and neural tissue injury induce the symptoms of a sympathetic storm, accompanied by a massive increase in catecholamine levels and, secondarily, increased myocardial metabolism, followed by adverse changes in myocardial contractile and Takotsubo cardiomyopathy [15,16,17,18,19]. The value of high-sensitivity troponins was elevated in 65% of the TBI cases, creatine kinase was elevated in 93% of the cases, and all were associated with adverse neurological prognoses. TBI patients with elevated troponin levels were significantly more likely to require intubation for airway protection and longer mechanical ventilation support during hospitalization, more frequently report pneumonia and sepsis, and require longer ICU hospitalization [20,21,22,23]. Although there are markers available for myocardial damage, it is also crucial to identify universal markers indicating the links between multi-organ dysfunction, including primary brain damage and secondary myocardial failure.

Oxidative stress is a common cellular dysfunction responsible for the pathogenesis of primary acute traumatic brain injury and secondary organ dysfunction. Oxidative stress results from the imbalance between the production of reactive oxygen species (ROS)/nitrogen species (RNS) and the impairment of antioxidant defenses, which disrupts gene expression and cell metabolism. The brain tissue is highly susceptible to oxidative damage due to its high oxygen consumption, low antioxidant capacity, and strictly limited regeneration mechanisms [24,25]. The disruption of the blood–brain barrier (BBB) causes redox imbalance to become systemic. Thus, oxidative stress, initially localized to the brain tissue, spreads to involve peripheral organs and tissues [26,27,28,29]. Disturbances to redox homeostasis represent a crucial pathophysiological element of acute primary and secondary critical organ failure, as well as chronic conditions and diseases such as atherosclerosis, diabetes mellitus, obesity, vascular malformations, and renal failure [30,31,32]. The endogenous sources of ROS/RNS include the mitochondrial electron transport chain, NADPH (NOX) oxidases, xanthine (XO) oxidases, endothelial nitric oxide synthase (eNOS), cytochrome p450, or cyclo-oxygenases (COX) [26,27,28,29,33,34,35,36]. Among all the ROS, superoxide radical anion (O_2_^−^) is produced in the largest quantities. Its formation occurs in the mitochondria via the NADPH–ubiquinone oxidoreductase complex, and O_2_^−^ can then be converted to non-radical hydrogen peroxide (H_2_O_2_). In the presence of iron or copper anions, H_2_O_2_ generates, via the Fenton reaction, the highly reactive hydroxyl radical (HO). HO can react with NO to form ONOO^−^, which is one of the strongest in vivo oxidizing agents. Free radicals and other ROS/RNS cause oxidative damage to proteins, lipids, and DNA, such as the formation of crosslinks and carbonyl groups, the peroxidation of cell membranes, and the mutation of genetic material, which directly contribute to tissue and organ damage [37,38,39,40,41,42]. Measuring damage due to ROS and RNS is a difficult task. The direct quantification of reactive species offers a promising biomarker; however, measurements are complicated by their short half-lives. Electron spin resonance, fluorescence, and magnetic resonance techniques are limited to only cell cultures. More popular methods focus on determining the products of lipid and protein oxidation—such as malondialdehyde (MDA), isoprostanes (F2-IsoPs), alkenals, alkadienals, 4-hydroxy 2-nonenal (4-HNE), advanced glycation end products (AGEs), protein carbonyls, and advanced oxidation protein products (AOPPs) as well as the products of nucleic acid modifications (8-oxo-2’-deoxyguanosine)—based on gas/liquid chromatography, mass spectroscopy assays, immunodetection using ELISA tests, and colorimetric methods [43,44,45,46,47,48,49].

In addition to the biomarkers of oxidative stress, assessments of the antioxidant barrier are also used in clinical practice. Antioxidants play a crucial role in protecting cells against the harmful effects of ROS and RNS. The activity of enzymatic antioxidants (superoxide dismutase (SOD), catalase (CAT), and glutathione peroxidase (GPx)), the concentration of non-enzymatic antioxidants (glutathione (GSH), bilirubin, uric acid, albumin, lactoferrin, vitamins C and E, coenzyme Q, and lipoic acid), and trace elements, such as selenium, are the main indicators determining antioxidant status [17,18,19,20,21,22,50,51]. However, due to a plethora of antioxidants and ROS/RNS, total antioxidant capacity (TAC) and total oxidant capacity (TOC) are increasingly used in clinical practice. Total antioxidant capacity refers to the number of moles of a given oxidant scavenged by the sample studied. The idea of measuring the TAC with a single assay seems attractive because this method is less troublesome and cheaper compared with the measurements of numerous individual antioxidants, and it does not require sophisticated equipment. These assays have become popular, have been commercialized, and have a chance to become standard clinical laboratory tests [52,53,54,55]. Samples such as blood (serum and plasma), urine, saliva, and cerebrospinal fluid (CSF) are the most popular biofluids used for TAC examination [6,23,56,57,58,59,60,61,62,63,64,65,66].

The total oxidant capacity (TOC) refers to the cumulative effect of both enzymatic and non-enzymatic antioxidants in counteracting oxidative stress by neutralizing reactive peroxides. It determines the concentration of peroxides (H_2_O_2_) based on Fenton reactions and colorimetric readings [12,13,14,15,16,30,31,32,67,68,69,70]. The relationship between oxidants and antioxidants can be represented by the oxidative stress index (OSI), calculated as follows: OSI = TOC (μmol H_2_O_2_ equivalent/L)/TAC (μmol Trolox equivalent/L) × 100 [67,71].

At present, it is important to identify a universal marker of redox balance that will predict the clinical prognoses of critically ill patients and their organ function [2,33,57,72,73]. In this review article, we summarize the current state of knowledge about the antioxidant–oxidant status in critically ill patients with traumatic brain injury (TBI). In addition, the aim of this review was to present the diagnostic value of TAC, TOC, and OSI in predicting secondary organ dysfunction, neurological complications, and mortality.

## 2. Materials and Methods

The aim of this review was to describe biomarkers for oxidant–antioxidant status in traumatic brain injury and correlate them with radiological and clinical stages, neurological prognostication, and markers of secondary organ failure. The inclusion and exclusion criteria are presented in Table 1. A literature search was conducted up to 1 September 2024, according to the Preferred Reporting Items for Systematic Reviews and Meta-Analyses (PRISMA 2020) guidelines, using the PubMed, Web of Science, and Scopus databases. Only international publications written in English were evaluated.

The available literature was browsed based on the following keywords: total oxidative capacity and TBI, total antioxidative capacity and TBI, oxidative stress and TBI, redox and TBI, TAC and brain trauma, TOC and brain trauma, oxidative index and brain trauma, redox status and brain trauma, plasma oxidation–reduction and brain trauma, TAS and brain trauma, TOS and brain trauma, plasma total oxidative capacity and brain injury, plasma total antioxidant capacity and brain injury, cerebrospinal TOC and brain trauma, and cerebrospinal TAC and brain trauma.

Data pre-selection was carried out independently by two authors based on the titles and abstracts. The publications that met the inclusion criteria were considered for the review. The reliability level was determined by Cohen’s kappa coefficient, which was 0.92. All the publications were analyzed for methodology, year of publication, study design, sample size in the study group, and results.

According to the Oxford Center for Evidence-Based Medicine (CEBM) 5-level classification scale of diagnosis, most of the studies presented in the third level of evidence (clinical cohort studies).

According to the Study Quality Assessment Tool guidelines issued by the National Heart, Lung and Blood Institute (NHLBI) within the U.S. National Institutes of Health (NIH), a critical assessment was conducted.

## 3. Results

**Figure 1:**
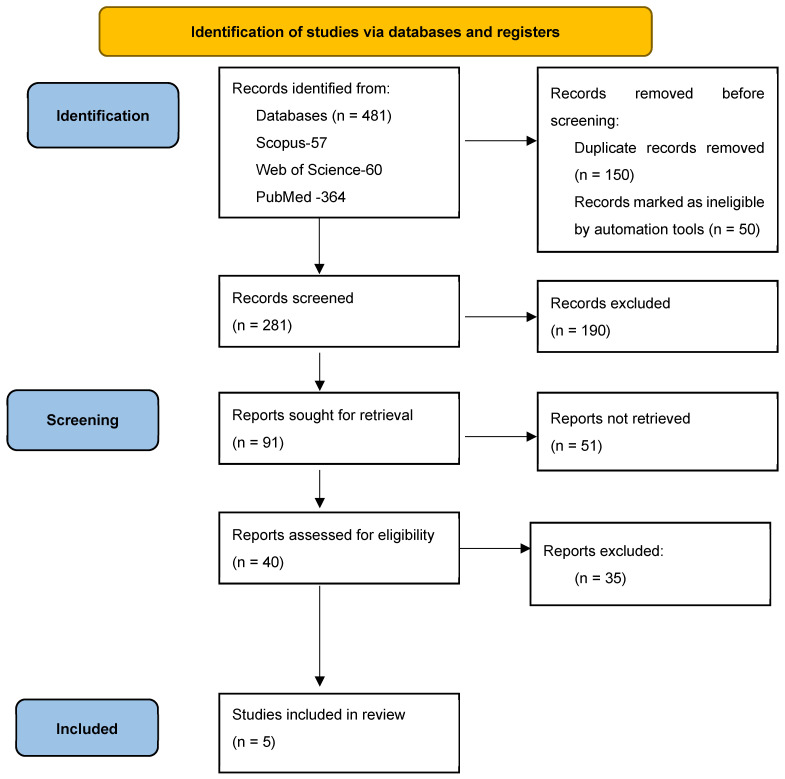
PRISMA flow chart.

The literature search revealed 364 articles from PubMed, 57 articles from Scopus, and 60 articles from Web of Science. The PRISMA flow diagram is presented in Figure 1. Finally, only those that reported exclusively TAC and TOC in brain trauma are presented in Table 2.

TAC was analyzed as a clinical predictor of neurological condition and mortality. A TAC in the CSF above 300 µmol/L was associated with 95.5% of good neurological prognoses, with a mean hospitalization time of 34 days. In contrast, a serum TAC concentration of ≥450 µmol/mL showed 72% sensitivity and 52% specificity, with an AUC of 0.662 for good prognostication. Moreover, a TAC level in the CSF of ≥300 µmol/L indicated 70% sensitivity and 50% specificity, with an AUC of 0.577 for good neurological outcomes [23].

The 30-day mortality rates based on TAC levels exceeding 5.60 mmol/L on day 1 corresponded to a sensitivity of 59%, specificity of 93%, NPV of 86%, and PPV of 77%; on day 4, the sensitivity increased to 82%, specificity was 76%, NPV was 96%, and PPV dropped to 39%; on day 8, the sensitivity was 69%, specificity was 93%, NPV was 95%, and PPV was 60%. The study showed that the TAC concentration was the best predictor of 30-day mortality determined on the fourth day after TBI [48].

Another study noted that a serum TAC of >2.59 nmol/mL was an indicator of 30-day mortality with an AUC of 0.83, sensitivity of 78%, specificity of 63%, PPV of 44%, and NPV of 66%. The TAC values correlate with mortality rate based on the Glasgow Coma Scale, CT-based radiological signs, and pupil reactivity. The plasma oxidation–reduction potential is a better predictor of mortality than the clinical scales of injury and neurological condition [43].

## 4. Discussion

The search for specific substances in fluids, cells, and tissues responsible for the occurrence of disease processes is a very current trend in medicine. Publications in recent decades have pointed out the multidirectional effects of oxidant–antioxidant processes on the maintenance of homeostasis. An ideal biomarker of oxidative stress in TBI should be highly specific for brain tissue, non-invasive in its acquisition, simple in its technical laboratory assays, and stable under various environmental conditions. Moreover, the biomarker of cerebral trauma should present a high diagnostic value in predicting the clinical consequences of the injury—the neurological condition, the need for surgical intervention, and advanced brain imaging and monitoring [74,75,76]. A wide variety of methods (inhibition and reduction assays) have been proposed for TAC estimation. Generally, 2-amidopropane (ABAP) is the source of oxidizing radicals in inhibition methods. Spectrophotometric or fluorometric detection is based on the chromogenic substrate 3-ethylbenzthiazoline-6-sulfonic acid (ABTS). The total antioxidant status (TAS) introduced by Randox measures the inhibition of ABTS oxidation in a system of metmyoglobin and hydrogen peroxide. The oxygen radical absorbance capacity (ORAC) is based on the bleaching of the fluorescence of phycoerythrin or allophycocyanin (fluorescein) by ABAP and the protection of the oxidation substrates by antioxidants. The reduction methods of TAC assays are based on the assumption that antioxidants act as reductants, with the number of components capable of reducing an indicator reflecting the number of antioxidants present in the sample. The DPPH reduction method uses 1,1-diphenyl-2-picrazyl (DPPH) radicals. The ferric-reducing antioxidant potential assay (FRAP) measures the reduction of a ferric complex of 2, 4, 6-tripyridyltriazine to a colored ferrous complex. ABTS^+^ decolorization is also used in the reduction methods of TAC assays, with absorbance at 414 nm. TAC determinations result from the complex interplay of antioxidant compounds, simple thiols, and uric acid groups; therefore, there is a high risk of clinical misinterpretation within any condition associated with the elevated concentrations of these substances. The TAC value is calculated from standard curves for 6-hydroxyl-2, 5, 7, 8 tetramethylchroman-2-carboxylic acid (TROLOX equivalents (mmol, μmol)) and is expressed as the Trolox equivalent antioxidant capacity (TEAC). This represents the millimolar concentration of TROLOX with the same antioxidant capacity as a 1 mM solution of the compound under study [71,77,78,79,80,81,82,83,84]. TOS determination is performed through the reaction of peroxidase with peroxides in the sample, followed by the conversion of 3, 3, 5, 5 tetramethylbenzidine to a colored product, and absorbance is measured at 450 nm. The TOS is expressed in μmol H_2_O_2_ equivalent/l. TOC determinations are widely used methods to represent the complexity of oxidative processes [12,13,14,15,16,30,31,32,67,68,69,70].

### 4.1. Brain Injury

The available literature does not clearly indicate the utility of redox biomarkers in predicting TBI prognosis. We found only four original articles that presented the utility of TAC in TBI prognostication and only one original article that described plasma oxidation–reduction potential. We did not find any original articles about TOC in TBI.

Publications on acute neurological and neurosurgical conditions have confirmed that the serum TAC concentrations in brain ischemic stroke remain significantly higher in nonsurvivors compared with survivors, with serum TAC values of 5.33 mmol/mL vs. 2.38 mmol/mL (*p* < 0.001) and MDA values of 2.93 nmol/mL vs. 1.90 nmol/mL (*p* = 0.004), respectively. A correlation between serum TAC and MDA was also confirmed (rho = 0.35, *p* = 0.008). TAC was identified as a good predictor of general mortality, with an AUC of 0.82 (*p* < 0.001). TAC values of more than 3.39 mmol/mL indicated a sensitivity of 72%, a specificity of 79%, a positive predictive value (PPV) of 75%, and a negative predictive value (NPV) of 73% in predicting mortality. In addition, serum TAC values of more than 3.39 mmol/mL were linked to significantly higher 30-day mortality [43,44]. It was also described that on days 1 and 7 after ischemic stroke, significantly reduced concentrations of serum TAC were observed [44].

Redox biomarkers have also been described in TBI with sub-arachnoid hemorrhages. The mean plasma TAC did not change to the point of statistical significance during observation between day 1, day 3, and day 7 (77.8, 92.64, and 74.07 µmol/L, respectively), although it became statistically lower than the controls on days 3 and 7, for which these values were 177.3 µmol/L and 85.35 µmol/L. Poor short-term (6 weeks) and long-term (6 months) neurological outcomes were correlated with an increased plasma TAC. The total antioxidant activity in the cerebrospinal fluid showed no significant correlation with the neurological state. Moreover, decreased SOD in the CSF and plasma was associated with poor neurological outcomes at 6 weeks and 6 months [45,46,47].

Redox homeostasis was also described using other biomarkers. In analyzing the dynamics of oxidative stress based on products of lipoxygenation and protein carbonylation (TBARSs and protein carbonyls), it was found that serum TBARS levels were significantly higher 12 h, 30 h, and 70 h after TBI compared with a healthy control group, and the highest concentration was recorded at 70 h after TBI. The highest concentrations of carbonyls were recorded at 30 h after TBI, while their concentration at 70 h was significantly lower than at 30 h but also significantly higher than at 12 h. In addition, there was no significant statistical difference between predicting mortality based on TBARS and carbonyls over any time period. No correlation was indicated between the concentrations of oxidative stress biomarkers and the advancement of post-traumatic lesions according to Marshall’s classification, nor was there any correlation with sub-arachnoid hemorrhage prediction based on the biomarkers indicated. The significant factors determining a poor prognosis were a pH of >7.35 (*p* = 0.01), serum glucose concentrations of >150 mg/dL (*p* = 0.01), serum sodium concentrations of >144 mmol/L (*p* = 0.04), hematocrit of >31 (*p* = 0.02), and hemoglobin level of >11 (*p* = 0.03) [42].

Redox status analyses immediately after TBI indicated that the SOD activity in the erythrocytes (1947 U/g Hb) was significantly lower in patients after TBI compared to a control group (2849 U/g Hb) (*p* = 0.001), while their TBARS concentrations were non-significantly higher (13.62 µmol/L vs. 12.52 µmol/L (*p* = 0.072)) compared to the control group. The group of patients with a poor prognosis after TBI had significantly lower GSH concentrations and significantly reduced erythrocyte SOD activity on day 1, while the observations on days 4 and 7 showed no characteristic changes in the concentrations of these biomarkers. Poor prognostic factors were connected with clinical and radiological symptoms (GCS classification, brain injury on CT scans, motor deficits, unconsciousness, neurosurgical interventions, and postoperative complications), as well as serum GSH levels and erythrocyte SOD activity [85].

An evaluation of antioxidant status based on thiol group concentration showed that the concentration was significantly lower among TBI patients compared to healthy controls, at 210 µM vs. 301 µM (*p* < 0.0001). In a mild TBI group, the concentration of free thiols in the serum was 225.9 and was higher than that in moderate TBI and severe TBI groups, which saw levels of 209.7 and 199.2 µM, respectively. In addition, hypoxia and hypotension significantly reduced the thiol concentration (*p* < 0.001). In the cases of complex injuries, the thiol concentrations reached significantly lower values in TBI, at 186 µM vs. 231 µM, respectively (*p* = 0.01). However, no correlation was indicated between thiol concentrations and the severity of traumatic lesions in TBI based on CT scans and neurological status, as assessed by the GCS. Equally, the recovery of thiol substances was noted after 6 months in patients with a good neurological status after TBI [86].

### 4.2. Organ Dysfunction in the Course of TBI

It was shown that the plasma levels of the oxygen free radicals O_2_^−^, H_2_O_2_^−^, and NO_2_^−^, and TBARSs were significantly higher in a group of patients with sepsis compared with a control group, at 1.93 nmol/mL, 2.95 nmol/mL, 2.71 nmol/mL, and 1.17 µmol/mL, respectively. At the same time, the mean SOD, CAT, and GSH concentrations in erythrocyte hemolysate were significantly lower (25 U/g Hb × 10^3^, 2.35 U/g Hb × 10^3^, and 99,125 U/g Hb × 10^3^, respectively), confirming the predominance of oxidative processes. In addition, a strong correlation was indicated between recognized inflammatory indicators—namely C-reactive protein (CRP), procalcitonine (PCT), and white blood cells (WBCs)—and redox parameters (*p* < 0.05). Moreover, the statistical analysis showed a positive correlation between ROS levels in the form of O_2_^−^ and H_2_O_2_ and the parameters of the WBC count, CRP, and PCT and a negative correlation between the antioxidant parameters of SOD and CAT and WBCs, CRP, and PCT (*p* < 0.05). NOS was also correlated with the parameters of DNA damage (the genetic damage index (GDI)) and CRP. The exponents of DNA damage reflected in the GDI were higher to the point of statistical significance in the patients with sepsis, reaching minimum values of 1.64 to 2.58 and indicating moderate damage. The GDI showed a correlation with inflammatory parameters and ROS and NOS levels. Further analysis indicated that the prognostically unfavorable factors in sepsis were age, O_2_^−^ and CRP concentrations, and the GDI [83]. At the same time, significantly higher serum MDA concentrations were noted among the nonsurvivors of sepsis. Serum TAC was associated with 30-day survival (hazard ratio = 1.50; 95% CI = 1.16–1.94; *p* = 0.002), as were APACHE II (1.04, 95% CI = 1.01–1.08, *p* = 0.02), lactate concentration (1.08, 95% CI = 1.01–1.15, *p* = 0.009), and renal failure rates (1.61, 95% CI = 0.94–2.78, *p* = 0.08). ROC analysis presented that the AUC for TAC in predicting 30-day mortality was 0.61 (*p* = 0.04), with a sensitivity of 21%, a specificity of 89%, a positive predictive value of 52%, and a negative predictive value of 69%. It was also indicated that TAC concentrations above 2.8 mmol/L accounted for lower odds of 30-day survival. No correlation was shown between TAC and MDA [84].

Other researchers have highlighted differences in the methodologies used to determine TAC depending on the biological material used and the time of their collection in relation to hospitalization. Discrepancies in serum and plasma TAC concentrations can be explained by the presence of uric acid (UA), which accounts for nearly 60% of plasma’s total antioxidant capacity. Long-term observations indicated that the highest TAC concentrations were recorded during the first few hours of hospitalization for critically ill patients with sepsis and that their TAC concentrations decreased significantly on subsequent days [71,82,83,84].

By using serum-derived reactive oxidant metabolites (dROMs) as the oxidative index and biological antioxidant potential (BAP) as the antioxidative index, Izumino et al. assessed the redox status and its correlation with mortality in critically ill patients. In addition, these authors used a modified ratio (MR) mathematically calculated as BAP/dROMs/7.51, where an MR value of <1 indicated oxidative stress. In the study group, 42% were trauma victims, and the survivors had higher dROM values and lower BAP values compared to the nonsurvivors. Although there was no difference in the dROM values between the survivors and healthy controls, significantly lower dROM concentrations were observed in the nonsurvivors. The BAP in the nonsurvivors was significantly higher and correlated with mortality. Antioxidant potential, as assessed by BAP (nonsurvivors: 2852; survivors: 2137; healthy volunteers: 2320), and an increased MR were the determinants of mortality, with medians of 1.507 for nonsurvivors, 0.924 for survivors, and 0.943 for healthy volunteers. In addition, the dynamics of the changes in the BAP and MR were described as increasing mortality proportionally. The ROC analysis elucidated that the AUCs for dROMs, BAP, and MR were similar, at 0.66, 0.80, and 0.82, as well as for the APACHE II scale, at 0.87, respectively. The MR was most closely related to mortality outcomes. Moreover, combining BAP with the APACHE II scale had the greatest predictive value for mortality in critically ill patients [67].

## 5. Limitations

To our knowledge, this study is the first systematic literature review comparing the different biomarkers of redox status in traumatic brain injury and secondary organ failure. Unfortunately, the study protocol was not registered, and due to the high heterogeneity of the studies, we could not use advanced statistical methods to analyze the data, which would have increased the quality of the conclusions. The inclusion and exclusion criteria of the search were precisely specified, and only a small number of articles were found. Additionally, articles about TAC, TOC, and OSI in chronic disorders were not analyzed.

The analysis of the methodologies in the available publications indicated that the study population sizes were relatively small but precisely defined the inclusion and exclusion criteria. However, the inclusion criteria varied based on the types of neurosurgical interventions and classifications of neurological condition (GCS), trauma severity (ISS), and general health status (APACHE II).

In addition, a variety of biochemical methods were used to assess the redox status, making objective data comparison difficult. Therefore, future studies should include randomized clinical trials on larger populations of patients with TBI.

Moreover, cohort studies and meta-analyses are necessary to accurately describe the significance and clinical application of TAC and TOC assays. The articles presented in Table 2 refer to the determination of TAC and TOC in the acute post-traumatic phase (1–7 days), and the 30-day mortality was compared to the first determinations. Undoubtedly, a prolonged analysis of TAC and TOC changes could provide greater predictive value in TBI prognosis.

## 6. Conclusions and Future Directions

Many studies have widely substantiated the utility of biomarkers for assessing the severity and prognosis of chronic conditions, such as renal failure, heart failure, diabetes mellitus, dental conditions, psychiatric and neurodegenerative disorders, alcohol and nicotine abuse, and rheumatologic conditions [30,31,32,62,63,64,68,69,70]. Moreover, oxidative stress is a significant pathogenic element of carcinogenesis [65,66], and oxidant–antioxidant imbalance has also been described in the course of opioid and anti-inflammatory drug therapies [24,25]. Of course, the greatest expectations arise when describing the biomarkers of oxidative stress in the prognosis of life-threatening conditions and long-term disability, including central nervous system injuries [87,88,89,90,91].

The conducted analysis process highlighted the limited number of publications on oxidative stress based on TAC and TOC measurements in TBI diagnostics and prognosis. The presented articles reported TAC and TOC assays in serum, plasma, and cerebrospinal fluid. The determinations of TAC and TOC in other biological materials such as urine, saliva, and bronchoalveolar lavage with different analytical methods represent the future perspective in the diagnosis of brain condition, as well as other organ functions.

## Figures and Tables

**Table 1:** The inclusion and exclusion criteria.

Exclusion Criteria	Inclusion Criteria
- Publications written in any language other than English; - Case reports, abstracts, interventional studies, and review articles; - Experimental studies based on animals; - Clinical studies performed on a group of fewer than 20 adult patients; - Materials published before 2005.	- Articles written in English; - Original observational and clinical studies conducted on a group of at least 20 adult patients with a traumatic brain injury; - Articles published after 2005.

**Table 2:** A summary of the results of the systematic review.

Authors	Publication	Type of Study and Sample Size	Methods, End-Point	Results
Muballe et al. [23]	*Journal of Neurosurgery* 2019	prospective study *n* = 64	serum and CSF TAC; FRAP day 1–7–14, neurological recovery	TAC in the CSF above 300 µmol/L was accounted for 95.5% of good neurological prognoses, with a mean hospitalization time of 34 days. Serum TAC ≥ 450 µmol/mL accounted for 72% sensitivity, 52% specificity, and AUC 0.662, TAC in the CSF ≥ 300 µmol/L equated to 70% sensitivity, 50% specificity, and AUC 0.577.
Servia et al. [74]	*PlosOne* 2018	prospective cohort study *n* = 66	plasma TAC; comparing TAC based on FRAP and ABTS, at days 1–7 correlation with clinical scales	TAC values on day 1 based on FRAP were significantly lower in TBI—919 µM TE; on day 7, there was a TAC increase for the TBI. There were no significant differences in the TAC values between the groups on either day 1 or day 7 using the ABTS method. A positive correlation between uric acid and TAC using the FRAP method and a negative correlation between bilirubins and TAC using the ABTS method were noted. A negative correlation of the APACHE II scores with TAC and a positive correlation of the GCS classification with TAC according to FRAP.
Lorente et al. [47]	*Brain Science* 2020	multicenter, observational, prospective study *n* = 124	serum TAC; ABTS; day 1–4–8; 30-day mortality	TAC was significantly higher in the nonsurvivor group on day 1, 5.60 mmol/L; on day 4, 4.43 mmol/L; and on day 8, 3.29 mmol/L. On day 30 after a TBI, the median TAC was 5.60 mmol/L. The 30-day mortality rates on day 1 corresponded to a sensitivity of 59%, a specificity of 93%, an NPV of 86%, and a PPV of 77%; on day 4, a sensitivity of 82%, a specificity of 76%, an NPV of 96%, and a PPV of 39%; on day 8, a sensitivity of 69%, a specificity of 93%, an NPV of 95%, and a PPV of 60%
Lorente et al. [75]	*BMC Neurology* 2015	multicenter, observational, prospective study *n* = 100	serum TAC; ABTS; 30-day mortality; correlation with clinical scales	TAC values in patients with brain injuries were significantly higher in nonsurvivors (5.09 nmol/mL) than in survivors (2.31 nmol/mL). Serum TAC > 2.59 nmol/mL is an indicator of 30-day mortality, AUC of 0.83, a sensitivity of 78%, a specificity of 63%, a PPV of 44%, and an NPV of 66%. TAC is compared with mortality parameters based on GCS scores, age, CT-based radiological signs, and pupil reactivity.
Bjugstad et al. [76]	*Oxidative Medicine and Cellular Longevity* 2016	retrospective cohort study *n* = 132	plasma oxidation–reduction potential (ORP); RedoxSys; day 1–2–7 weeks 2, 3, 4 surviving and severity of acute brain injury	Predicting acute outcomes—ORP ≥ 7.25 µC on day 4, 100% specificity, a positive predictive value of 97%, and AUC 0.87. A better predictor of mortality than clinical scales of injury ISS, AIS, and GSC.

## Data Availability

Data are contained within the article.
